# Can Engagement Go Awry and Lead to Burnout? The Moderating Role of the Perceived Motivational Climate

**DOI:** 10.3390/ijerph16111979

**Published:** 2019-06-04

**Authors:** Christina G. L. Nerstad, Sut I Wong, Astrid M. Richardsen

**Affiliations:** BI Norwegian Business School, 0442 Oslo, Norway; sut.i.wong@bi.no (S.I.W.); astrid.richardsen@bi.no (A.M.R.)

**Keywords:** work engagement, burnout, mastery climate, performance climate, well-being

## Abstract

In this study, we propose that when employees become too engaged, they may become burnt out due to resource depletion. We further suggest that this negative outcome is contingent upon the perceived motivational psychological climate (mastery and performance climates) at work. A two-wave field study of 1081 employees revealed an inverted U-shaped relationship between work engagement and burnout. This finding suggests that employees with too much work engagement may be exposed to a higher risk of burnout. Further, a performance climate, with its emphasis on social comparison, may enhance—and a mastery climate, which focuses on growth, cooperation and effort, may mitigate the likelihood that employees become cynical towards work—an important dimension of burnout.

## 1. Introduction

Work engagement, defined as a positive and fulfilling state of mind related to work [1], has been shown to be associated with desirable individual and organizational outcomes such as better health, high work performance, shareholder return, profitability, customer satisfaction, and low absenteeism and turnover (e.g., [2,3,4,5]). Work engagement has typically been described as a means to enable a situation in which both employees and employers benefit [6]. This universally positive view of work engagement has been challenged, and the investigation of alternative interpretations, including a dark side of engagement, has been called for (e.g., [6,7]). 

The idea of the downside of work engagement is based on the premise that too much engagement can lead to over-engagement in work-related tasks. According to the conservation of resources theory (COR) [8], individuals continuously pay attention to the amount of resources they have available, and they seek to acquire and maintain their resources (e.g., energies, objects, conditions, and personal characteristics) when they are threatened. In line with COR, engaged employees increase their positive resources at work through a so-called “gain spiral” in which resources beget more resources [9]. However, overly engaged employees may end up with a higher workload than those who are less engaged [10,11], which makes them more likely to deal with increased job demands by working longer hours and nearing or exceeding their limits [11]. Accordingly, employees 1engagement may be curvilinearly related to burnout such that at particularly high levels, engagement can be a burden rather than a buffer that prevents job burnout [12,13]. By drawing on the meta-theoretical principle of the too-much-of-a-good-thing (TMGT) [14] effect—meaning that favorable predictors reach inflection points and their associations with outcomes discontinue to be linear—a few studies have found support for a curvilinear relationship between engagement and outcomes such as turnover intention, psychological distress and job performance [13,15]. Still, scholars have called for more research to clarify the curvilinear association between work engagement and health-related outcomes (e.g., [13]). Therefore, our study’s first purpose is to investigate the alleged curvilinear relationship between work engagement and burnout. This is important, as it may increase our understanding of the benefits and potential costs of work engagement for employees and their organizations. 

Research also points to the influence of work situations on both work engagement and burnout [16,17,18]. This study will examine the extent to which one such environmental factor, the perceived motivational psychological climate, influences the relationship between work engagement and burnout. According to the achievement goal theory (AGT), the motivational climate—or the perceived criteria of what constitutes success and failure in the work situation—can focus on either mastery or performance [19,20]. A mastery climate emphasizes the value of employee effort, self-development, and cooperation [19]. The focus is on the process rather than on the end results, which may help employees reallocate and retain their resources (e.g., autonomy or relatedness) to protect themselves from strain and burnout [21]. In contrast, a performance climate values social comparison and competition between employees [19]. Employees in such a climate may use most of their resources to focus on this competition, resulting in a reduced ability to cope with a high number of job demands. Assuming that the motivational climate has a moderating influence on the potential curvilinear relationship between engagement and burnout, our study’s second purpose is to gain insight into the contextual mechanisms that may play a role in that relationship.

Our study offers two key theoretical contributions to the occupational health psychology literature. First, the downside of work engagement has been inadequately researched [12,13] and the existing conceptualizations and research findings concerning work engagement and burnout are inconsistent [22,23]. Some argue that these concepts are at opposite ends of a continuum [24], others suggest that they are antipodal counterparts but distinct concepts [25], and still others advocate that they may not be independent phenomena [26]. A recent study and a review article showed that burnout and engagement are distinct, rather than being conceptual opposites, which emphasizes the importance of assessing burnout and engagement independently [22,27]. By testing a curvilinear relationship between work engagement and burnout, we aim to advance current knowledge and contribute to the engagement and burnout literature by clarifying the potential hazards of high work engagement in relation to burnout [7,13,28,29].

Second, by examining the moderating role of the perceived motivational climate, this study seeks a more contextualized understanding and a clarification of the person-situation interplay [30,31]. So far, research on the engagement-burnout relationship combined with the perceived motivational climate is scarce. This is unfortunate given that mastery and performance climates are important aspects of organizational life [32,33,34]. Mastery and performance climates may add a contextual element to the understanding of the engagement-burnout relationship by clarifying how employees are sensitive to and process salient contextual cues, goals, and values (cf. [23,31]). Differentiated contextual cue information processing may affect employees’ action strategies for these goals, which in turn may affect their well-being [35,36]. Our findings may also be helpful in developing effective motivational climate interventions in organizations that are particularly concerned with nurturing the work environment to capitalize on work engagement’s positive influence on well-being.

### Work Engagement and Burnout

Work engagement is defined as an affective and motivational state of work-related well-being that is persistent, positive and characterized by dedication, vigor, and absorption [7]. Vigor can be characterized by high levels of energy, dedication indicates a strong work involvement, and absorption characterizes a state of full concentration and happy devotion towards an activity [37]. Although these three engagement subdimensions can be theoretically distinguished, they are closely related [25,38]. 

Research has emphasized that work engagement facilitates positive individual and organizational outcomes, including, for example, a high degree of well-being, in-role and extra-role performance, profitability, and low absenteeism (e.g., [2,16,39,40]). However, there are also theoretical indications that excessive engagement can lead to negative outcomes, including burnout [12,13]. 

Burnout is typically defined as a psychological syndrome of exhaustion, cynicism, and reduced professional efficacy that involves a prolonged response to chronic interpersonal stressors at work [17]. Exhaustion refers to feelings of strain, particularly chronic fatigue, but does not necessarily involve other people as the main source of tiredness [41]. Cynicism refers to distancing and generally indifferent attitudes toward work [42]. Professional inefficacy reflects an employee’s perceptions of reduced competence and a lack of successful achievement and accomplishment at work [17]. Previous research supports this three-dimensional nature of the burnout syndrome [43,44].

Individuals who are overly engaged are more likely to be absorbed in their work and to take work home, neglecting their personal lives [7]. For example, Beckers et al. [45] found that highly engaged Dutch employees also worked more overtime. The resulting work-home interference may undermine employees’ recovery from stressful work lives, resulting in their reduced well-being [46,47]. In addition, a study by Sonnentag [48] revealed that highly engaged employees experienced an increase in job demands over time. The increased job demands may be due to supervisors’ preferences to assign work tasks to highly engaged employees because they are more likely to both accept the tasks and spend more time on them [47]. On the other hand, for some other highly engaged workers, the increased workload may be due to an intrinsic self-imposed demand. This may be because they tend to be so enthusiastic about their work that they look for additional tasks, thus increasing the likelihood of overwork [12,48]. As a result, they may accomplish more than is expected, which their supervisors view as a positive outcome [12]. A negative spiral may emerge as the supervisors ask such employees to take on even more tasks, eventually leading to negative outcomes, such as exhaustion. However, this is not in line with the current understanding of work engagement, which is said to lead to higher degrees of personal happiness and well-being [12]. These findings point to a mechanism in which engagement can be associated with lower employee resources and poorer health and well-being [3,48]. 

Maslach [12] proposed that the association between engagement and burnout might not be purely linear and that it might instead be represented by a curvilinear pattern. That is, too much work engagement may not necessarily serve as a buffer against job burnout, but instead as a burden. As suggested by Macey and Schneider [49] (p. 25), “there are limits on the pool of energy and resources available to employees”. Excessive engagement may not only apply to emotional exhaustion, which is a typical outcome of excessive job demands [41], but may also result in cynicism and professional inefficacy. Over-engaged employees may respond with cynicism as a means of creating defensive cognitive distance from work activities or people at work in an attempt to cope with work overload and the resulting exhaustion. Furthermore, it is likely that over-engaged employees would feel the need to work, such as responding to e-mails, calls, or other work duties on evenings, weekends, and holidays, thus aggravating feelings of overwork, work-nonwork conflicts, and stress e.g., [6,45]. Therefore, these over-engaged employees may feel that they have a lack of resources in doing their jobs, resulting in negative self-evaluations. In other words, over-engaged employees may experience a negative sense of who they are and what they do (i.e., professional inefficacy). 

In sum, according to engagement theory, being engaged can have positive benefits for individuals. However, in line with TMGT, excessive engagement can be detrimental because it may result in diminished recovery due to less time and energy to pursue nonwork interests. Thus, the relationship between work engagement and burnout may be curvilinear, such that very high levels of work engagement may result in higher levels of burnout. We therefore hypothesize:

**Hypothesis** **1.** *Work engagement is curvilinearly related to burnout, including (a) emotional exhaustion, (b) cynicism, and (c) professional inefficacy, in an inverted U-shaped curve.*

## 2. The Moderating Role of the Motivational Climate

Both job burnout and work engagement can be viewed as a result of the dynamic interaction between employees and their environments [6]. Work constitutes an achievement arena for employees who strive to succeed at their jobs [50]. The perceived motivational psychological climate at work, as defined by AGT, refers to employees’ perceptions of the extant criteria of success and failure, which are communicated through the policies, procedures, and practices of their work environment [51]. Such a climate consists of two goal-reward structures—a mastery climate and a performance climate—that define how employees are evaluated in relation to others and to a goal [19]. The perceived motivational climate reflects the goals and values that are important to the organization, how workers can attain those goals, and what it takes to be successful [35].

In a mastery climate, employees perceive that success is defined based on their own efforts, development, learning, and cooperation. Such a climate expects and rewards self-referenced criteria of success, and an important emphasis is to create the opportunity for each employee to develop his or her potential [51]. A performance climate represents a situation of “forced social comparison” [52] (p. 537) in which employees perceive that success is defined in terms of superior ability and performance relative to others. The criteria of success are other-referenced because normative comparisons and within-group competitions form the basis for rewards [51]. Goal attainment in such a climate depends on whether one is a “winner” or a “loser” [35].

### 2.1. Linear and Curvilinear Moderating Influence of a Mastery Climate

In line with the AGT, a mastery climate may serve as a buffer against burnout because its goal andvalue orientation concerns growth, choice, learning, and positive interdependence with peers or leaders [34]. In such a climate, employees are likely to believe that effort is valued (e.g., trying to accomplish job demands), and they may therefore develop cognitive-based strategies that help them to achieve self-referenced, rather than normatively referenced success [35]. An attributional focus of effort is fostered in employees [35], and the highly engaged employee may be better able to protect his or her resources and cope more effectively with job demands. Previous research has indicated that such a climate is important for tempering ill-being (e.g., burnout) perceptions (e.g., [53]). For example, both autonomy and social support—two aspects of emphasis in a mastery climate—have been found to decrease strain [54]. Furthermore, a mastery climate fosters mastery orientation [19], which has been shown to enable individuals to deal more adaptively with high job demands [55] and protect them from burnout [56]. 

Learning opportunities have also been found to decrease feelings of emotional exhaustion [57]. A mastery climate places value on meaningful learning, opportunities for self-directed learning, and self-referenced performance standards [19]. A focus on process instead of results may help employees feel that they have more control over what they can do, such as planning and allocating the resources that will enable them to achieve goals (cf. [57,58]) or to tackle demand accumulation [59]. Consequently, a mastery climate is likely to help employees with high feelings of energy, involvement, and enthusiasm to focus on sustaining positive outcomes (cf. [55,57,60,61]) and enabling more adaptive behaviors. Intrinsic motivation, subjective vitality, positive affective states, harmonious passion, perceptions of relatedness, work engagement, and autonomy were all enhanced by participation in a mastery climate ([51,62,63,64]; see [60] for a review).

**Figure 1:**
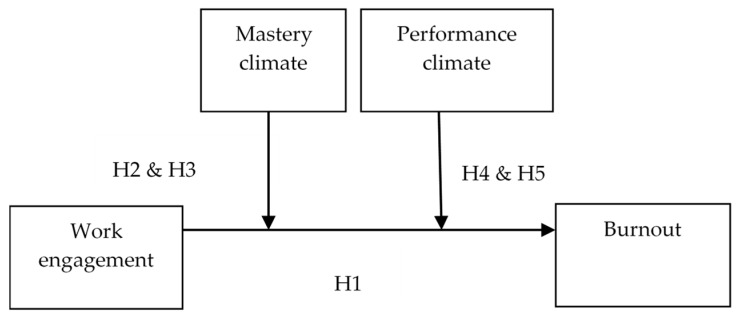
Theoretical model with hypotheses.

The posited curvilinear relationship between work engagement and burnout may be less likely to occur in a high-mastery climate than in a low-mastery climate. That is, the predicted inverted U-shaped curve is likely to be more flattened for employees who experience a high-mastery climate than for those who experience a low-mastery climate. We therefore hypothesize that a mastery climate moderates both the linear and curvilinear relationships between work engagement and burnout (Figure 1):

**Hypothesis** **2.** *A perceived mastery climate has a positive moderating role on the linear relationship between work engagement and burnout, including (a) emotional exhaustion, (b) cynicism, and (c) professional inefficacy. Specifically, this is demonstrated by an inflated linear slope of the curvilinear relationship for employees perceiving a high mastery climate.*

**Hypothesis** **3.** *A perceived mastery climate has a moderating role on the inverted U-shaped curvilinear relationship between work engagement and burnout, including (a) emotional exhaustion, (b) cynicism, and (c) professional inefficacy. Specifically, this is demonstrated by a flattening of the inverted U-shaped curve when the level of mastery climate is high.*

### 2.2. Linear and Curvilinear Moderating Influence of a Performance Climate

A performance climate may, on the other hand, enhance burnout among highly engaged individuals. Nicholls [65] (p. 133) stressed that “when winning is everything, you do anything to win!”. The inherent focus on outperforming other colleagues in a performance climate may be accompanied by a lack of concern not only for others [34] but also for oneself and one’s well-being [36]. Such a climate undermines important resources, such as autonomy, belonging, effort, self-referenced competence, skill development, and learning [62,66], and may make highly engaged employees experience a further loss of resources or believe that they have insufficient resources to cope with job demands [39,57]. Meta-analytical evidence suggests that individuals’ response patterns are likely to be maladaptive (e.g., burnout, performance anxiety) when a performance climate is salient [63]. For example, Lemyre et al. [56] found that elite winter sport athletes’ experiences of burnout were stronger in high-performance climate conditions.

We therefore suggest that a performance climate interacts with work engagement to enhance the likelihood that a highly engaged individual will experience burnout. The curvilinear influence of work engagement on burnout may be further exaggerated among employees who experience a high-performance climate, such that the inverted U-shaped curve becomes more dramatic. We therefore hypothesize that a performance climate moderates both the linear and curvilinear relationships between work engagement and burnout (Figure 1):

**Hypothesis** **4.** *A perceived performance climate has a negative moderating role on the linear relationship between work engagement and burnout, including (a) emotional exhaustion, (b) cynicism, and (c) professional inefficacy. Specifically, this is demonstrated by an attenuated linear slope of the curvilinear relationship for employees perceiving a high performance climate.*

**Hypothesis** **5.** *A perceived performance climate has a moderating role on the inverted U-shaped curvilinear relationship between work engagement and burnout, including (a) emotional exhaustion, (b) cynicism, and (c) professional inefficacy. Specifically, this is demonstrated by an exaggeration of the inverted U-shaped curve when the level of performance climate is high.*

## 3. Materials and Methods 

### 3.1. Participants

The sample included approximately 33,275 participants. The union’s registration system for members’ e-mail addresses is never completely up to date. Although the questionnaire was distributed to 33,275 addresses, the union received delivery failure e-mail messages from several of these; however, it did not keep a record of the number of delivery failures. Therefore, we cannot be certain about how many respondents received the surveys. Norwegian engineer and technologist union members representing different occupational divisions, such as research and development, human resource management, information technology, safety and the environment, health, consultancy, laboratory, sales and marketing, logistics, production, building and reconstruction, services, and economics. 

We received 8282 completed responses at Time 1 (T1), representing a response rate of approximately 25%. At Time 2 (T2), 4040 completed responses were received, representing approximately 49% of the T1 responses. Due to a technical problem with the web-based tool, it was only possible to match 1081 of the responses to respondents; therefore, we conducted an independent-sample *t*-test to examine possible differences between the 2959 respondents that we were unable to match and the 1,081 remaining respondents. According to the *t*-test results, there were some significant demographic differences in education, gender, and hours worked per week. However, there were no significant differences in the other study variables. We therefore controlled for the listed demographic variables in all analyses. 

In addition, we compared some of the demographic variables in our study to the union’s member-specific demographic statistics, which we were able to access. According to this comparison, which included age, gender, and work sector, the study participants seemed to be representative of the total union population, which includes approximately 66,000 members in total (mean age: 46.8 years; 78% male; 58% private sector; and 32% public sector). Of our total sample (T1), 75% were men, 53% worked in the private sector (compared to 32% in the public sector), and 85% had a university degree. Mean tenure in the present position was 3.35 years (standard deviation [SD]: 0.89) and the mean number of working hours per week was 40.45 (SD: 6.06).

### 3.2. Instruments

#### 3.2.1. Work Engagement

Work engagement was measured using the Norwegian version [67] of the Utrecht Work Engagement Scale (UWES-9 [25]) The scale consists of nine items (e.g., “When I get up in the morning, I feel like going to work”; “I am enthusiastic about my job”; “I am immersed in my work”) that are rated on a seven-point Likert-type scale, ranging from “never” (0) to “always/every day” (6). Cronbach’s α of the instrument varies from 0.89 to 0.97 [68]. Cronbach’s α in our study was 0.93 (T1) and 0.94 (T2). For more specific values concerning the validity of the scale see Schaufeli and Bakker [68] and Nerstad et al. [67].

#### 3.2.2. Burnout

We assessed burnout using the Norwegian version [43] of the Maslach Burnout Inventory-General Survey (MBI-GS [69]). We obtained permission to apply the scale through www.mindgarden.com. The items were represented by three subdimensions—emotional exhaustion (five items), cynicism (five items), and professional efficacy (six items)—and were rated on a seven-point Likert-type scale ranging from “never in the past year” (0) to “every day” (6). High scores on emotional exhaustion and cynicism and low scores on personal efficacy are indicative of burnout. Therefore, in this study, the personal efficacy items were reverse-scored [70]. One of the professional efficacy items (PE6) was omitted at T1 due to a computational error. However, upon inspection this omission did not seem to affect the results. 

A previous validation study found Cronbach’s α ranging from 0.57–0.91 for emotional exhaustion, 0.73–0.86 for cynicism, and 0.76–0.79 for professional inefficacy [71]. Cronbach’s α in our study was 0.87 for a mastery climate, 0.82 for cynicism, and 0.79 for professional inefficacy at T1, while it was 0.88 for emotional exhaustion, 0.82 for cynicism, and 0.78 for professional inefficacy at T2. For more specific values concerning the validity of the scale see Maslach et al. [69] and Richardsen and Martinussen [43,71].

#### 3.2.3. Perceived Motivational Climate

We measured the perceived motivational climate using 14 items developed and validated by Nerstad et al. [51]. The scale asks respondents how employees perceive that success is defined in their work situations. With eight items, the scale allows the respondents to assess the extent to which a performance climate (e.g., “In my department/work group, it is important to achieve more than others”) is present, and six items assess a mastery climate (e.g., “In my department/work group, one is encouraged to cooperate and exchange thoughts and ideas mutually”). The items were scored on a five-point Likert scale ranging from “strongly disagree” (1) to “strongly agree” (5). 

Previous studies found a Cronbach’s α ranging from 0.79–0.87 for mastery climate and 0.84–0.87 for performance climate (e.g., [34,72,73]). Cronbach’s α for a perceived mastery climate in our study was 0.85 (T1) and 0.87 (T2), while for a performance climate Cronbach’s α was 0.83 (T1) and 0.84 (T2). For more specific values concerning the validity of the scale see Nerstad et al. [51].

#### 3.2.4. Control Variables

Given the results of the *t*-test, we controlled for education, gender, and hours worked per week. Previous studies have found education to influence work engagement (e.g., [45]). Level of education was rated on a five-point scale: high school (1), vocational school (2), college degree (3), university degree (4), and other (5). Furthermore, gender has been found to impact work engagement, burnout, and perceptions of the motivational climate [74,75,76]. Gender was represented by a dichotomous variable: male (1) and female (2). Highly engaged employees have also been found to work longer hours [45], and the number of hours worked may affect burnout [41]. We also controlled for age, as research has shown that older employees are significantly more engaged than younger employees [74]. 

### 3.3. Procedure

The study design, planned sample, procedure, information letter to respondents, and questionnaires were evaluated and approved by Norwegian Social Science Data Services in order to ensure that high ethical standards were met. We conducted a two-wave study with a time interval of seven months. Consistent with COR theory, meta-analytical evidence indicates that the relationship between stressors and strains increases substantially over time, while the influence peaks after three years [77]. The development of burnout (i.e., strain) has no theoretically correct development pace, which according to Leiter [78] makes the choice of time lag an empirical question. Previous longitudinal research chose time lags varying between one month and one year [77,79]. Therefore, a time lag of seven months seems appropriate to test our hypotheses [77,78,80]. The time lag also facilitates the possibility of controlling for the relevance of time in the predicted relationships [77].

In order to adhere to its ethical standards, the union distributed the questionnaire to members through a web-based tool (QuestBack, Oslo, Norway). Along with the questionnaire, all respondents received an informational letter explaining the study’s background, how the data and personal information would be handled during the data collection process, and how the data would be anonymized. Furthermore, to make respondents less likely to edit their responses to make them more socially desirable, respondents were assured that the questions had no right or wrong answers and that they should respond to the questionnaire as honestly as possible. Thereby, we attempted to reduce common method biases (cf. [81]).

### 3.4. Data Analysis

To test the psychometric properties of the included scales and to facilitate control for discriminant validity [82], we first conducted a confirmatory factor analysis (CFA) on the T1 data applying the weighted least square with mean and variance adjustment (WLSMV) estimator for categorical data in M*plus* 7.3 [83,84]. We applied common guidelines to evaluate model fit (i.e., RMSEA < 0.08, CFI > 0.95 and TLI > 0.95 for an acceptable fit; [85,86]).

We further conducted descriptive analyses, reliability estimates, and bivariate correlations among the variables studied.

Next, using hierarchical linear modeling (HLM), we conducted multilevel regression analyses using SPSS 23 (IBM Corp, Armonk, NY, USA) to examine the within-person (i.e., T1 and T2) and between-person effects. HLM has been recommended for the analysis of data with repeated measures on the same individuals, which was the case in our study [87,88]. In the present study, the data are hierarchical in the sense that the measurement occasions are nested within individuals. This means that measurement occasions represent units of the first level and individuals represent units of the second level. An advantage of HLM is that differences among individuals with respect to the number of measurements (i.e., missing data) do not represent a problem [89,90]. Thus, the cases that only entail one measurement contribute less to the results of the two-wave regression since their data cannot be used to calculate the slope, but only the intercept of the multilevel regression [87,91]. Accordingly, we included T1 and T2 measures of all six variables studied in our analyses. That is, within each individual, there were two cases—corresponding to the T1 and T2 measures, respectively—producing a within-person equation and a between-person equation for each level of analysis. 

The interdependence test results revealed that the between-level variances for all six variables were significant, with *p* values less than 0.01. This implies that there is a considerable amount of variation for each of the six variables between individuals. We then conducted hierarchically moderated regression analyses to test our hypothesized curvilinear and moderating relationship [92]. Before computing the curvilinear and interaction terms, we centered the independent and moderating variables (work engagement, mastery climate, and performance climate). This was done to avoid the potential multicollinearity that can result from a correlation between curvilinear and/or interaction terms with main effects [93].

## 4. Results

Based on the CFA, a six-factor model assuming that all three engagement subdimensions load on the same factor reached acceptable fit (χ^2^ (650) = 32,973. 21, *p* < 0.001; RMSEA = 0.077; CFI = 0.922; TLI = 0.915). The inter-correlations between the engagement subdimensions were high (vigor and absorption: 0.75; dedication and absorption: 0.82), and particularly the correlation between vigor and dedication (0.92), which is in line with previous findings. We therefore decided to follow previous advice to use the total nine-item score (UWES-9) as an indicator of work engagement [25]. In addition, all factor loadings, except for one performance climate item (PC6: 0.41) and one professional inefficacy item (PE5: 0.46), were sufficiently high, ranging from 0.61 to 0.95, thus exceeding 0.50 [94]. Given that the scales have been previously validated, we decided to retain the two items in the further analyses.

As shown in Table 1, the coefficients of Cronbach’s α ranged from α = 0.79 to α = 0.93 at T1 and from α = 0.78 to α = 0.94 at T2, demonstrating satisfactory reliabilities for all scales measured. For the correlations among the six scales, the coefficients were at low to moderate levels. These results seem to support the findings of the CFA analyses, indicating empirical distinctness among the variables [82,95]. 

**Figure 2:**
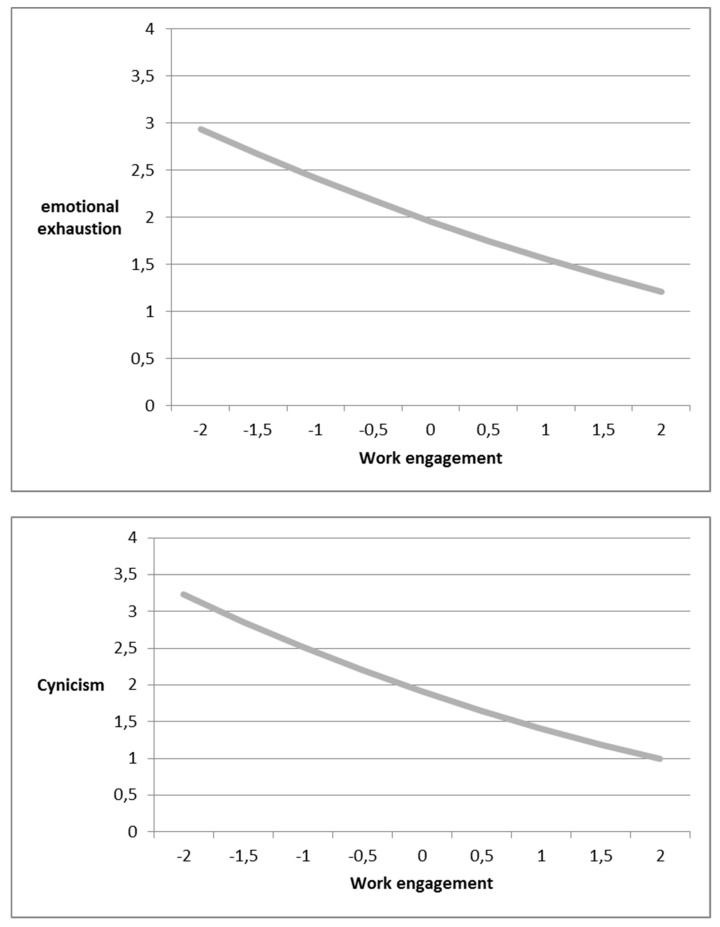
The curvilinear relationship between work engagement, emotional exhaustion, and cynicism.

To test the hypotheses, we first regressed (a) emotional exhaustion, (b) cynicism, and (c) professional inefficacy independently on the control variables (gender, age, education, and work hours). In the second step, work engagement and its quadratic (squared) term were added. As shown in Table 2, the results for emotional exhaustion, cynicism, and professional inefficacy all demonstrated a significant decrease in the deviance of the model after work engagement and its quadratic term were introduced. Moreover, work engagement appeared to be negatively related to emotional exhaustion (−0.43, *p* < 0.01), cynicism (−0.56, *p* < 0.01), and professional inefficacy (−0.45, *p* < 0.01). In addition, the results revealed that the curvilinear slopes between work engagement and the three subdimensions of burnout were significant. For emotional exhaustion (0.03, *p* < 0.01) and cynicism (0.05, *p* < 0.01), the coefficients of the squared work engagement were positive. Together with the negative linear slopes, the results indicate that emotional exhaustion and cynicism are higher when work engagement is lower and the levels decrease when the level of work engagement increases. However, the magnitudes of the two curvilinear relationships diminish when the level of work engagement continues to increase to a high level. To further inspect the forms of this relationship, we followed Aiken and West’s [93] procedure and plotted the graphs. As illustrated in Figure 2, the curvilinear relationships between work engagement and emotional exhaustion and between work engagement and cynicism demonstrated convex patterns, supporting Hypotheses 1a and 1b. However, for professional inefficacy, the coefficient of the squared work engagement was significant but negative (−0.02, *p* < 0.01). This result implies that the curvilinear relationship between work engagement and professional inefficacy has a U-shaped downward pattern. Hypothesis 1c was thus not supported.

**Figure 3:**
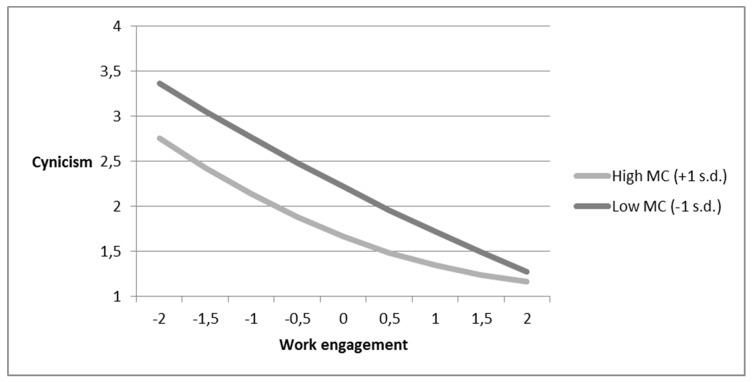
The moderating role of a mastery climate on the curvilinear relationship between work engagement and cynicism.

We further added mastery climate and its interaction terms with work engagement and squared work engagement to the model, along with performance climate as an additional control variable. The results, as shown in Table 2, demonstrate that mastery climate moderates the linear (0.08, *p* < 0.01) and curvilinear (0.03, *p* < 0.01) relationships between work engagement and cynicism. As depicted in Figure 3, employees who experienced a high level of mastery climate at work tended to report a lower level of cynicism. In addition, the negative influence of work engagement on cynicism was stronger for individuals who experienced a high-mastery climate when they had low-to-medium levels of work engagement. However, the negative influence became weaker when those individuals experienced medium-to-high work engagement. The results provided support for Hypotheses 2b and 3b. Unexpectedly, the mastery climate negatively moderates the linear (−0.03, *p* < 0.01) relationship between work engagement and professional inefficacy. However, the moderation of the curvilinear relationship did not yield significance. The moderating influence of the mastery climate was also insignificant for the linear and curvilinear relationship between work engagement and emotional exhaustion. Hypotheses 2a, 2c, 3a, and 3c were thus not supported.

**Figure 4:**
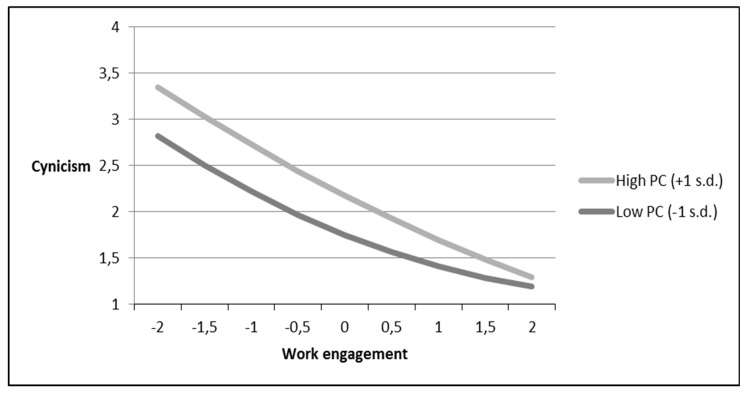
The moderating role of a performance climate on the curvilinear relationships between work engagement and cynicism.

Next, as we did for mastery climate, we added performance climate and its interaction terms with work engagement and with squared work engagement to the model while utilizing mastery climate as an additional control variable. As depicted in Table 2, performance climate negatively moderated the linear relationships between work engagement and emotional exhaustion (−0.07, *p* < 0.01) and between work engagement and cynicism (−0.08, *p* < 0.01), supporting Hypotheses 4a and 4b but not supporting Hypothesis 4c. However, performance climate only negatively moderated the curvilinear relationship between work engagement and cynicism (−0.02, *p* < 0.01). As illustrated in Figure 4, individuals who experienced lower levels of performance climate reported lower levels of cynicism. Moreover, the curvature of the relationship between work engagement and cynicism was stronger for those who experienced a low-performance climate, supporting Hypothesis 5b. However, the moderating influence of performance climate on the curvilinear relationship between work engagement and emotional exhaustion, and on the linear relationship between work engagement and professional inefficacy did not yield significance. Hypotheses 5a and 5c were thus not supported.

**Figure 5:**
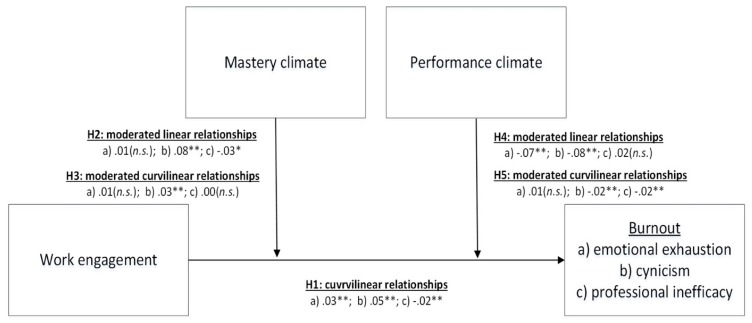
Theoretical model with hypotheses and results.

In Figure 5 we present the proposed model with the values from the analyses included.

## 5. Discussion

Drawing on the meta-theoretical TMGT effect principle [14], as well as the theoretical frameworks of COR theory [8] and AGT [19,65], we proposed that employees may be more vulnerable to burnout when their work engagement exceeds a certain level. We also suggested that this vulnerability is increased or inhibited depending on contextual contingencies—in this case, mastery and performance climates. First, our results indicate that being highly engaged at work may not be an exclusively positive factor due to a higher risk of burnout—in particular, emotional exhaustion and cynicism. Second, we found that a mastery climate may mitigate—and that a performance climate may enhance—the likelihood that employees will experience cynicism. 

### 5.1. Theoretical Contributions

Our study’s results represent two main theoretical contributions to the occupational health psychology literature. The first relates to work engagement theory and the common assumption that “more work engagement [is always] better” [96] (p. 253). Although some scholars have emphasized the need to consider the costs of work engagement for employees and organizations from a theoretical perspective, until now, no empirical research has clarified whether uniformly high levels of employee engagement could be detrimental with respect to burnout (e.g., [6,12]). Our results indicate that when employees are highly engaged, there is a potential for costs in the form of burnout vulnerability (i.e., TMGT effect principle). In line with the theoretical propositions of Maslach [97] and Maslach and Leiter [24], our empirical findings revealed that, when employees are highly engaged and expending high levels of energy, they seem to be more vulnerable to becoming affectively drained (i.e., emotional exhaustion) and wanting to alienate themselves from any meaningful involvement with other workers and other aspects of the job (i.e., cynicism). Thus, the theoretically assumed linear association between work engagement and burnout, which has been established in previous studies [3,42,98], may not capture the total complexity of the relationship between work engagement and job burnout. 

Furthermore, our results indicate that the theoretical supposition of engagement and burnout as opposites on a continuum may not be entirely accurate e.g., [24]. That is, the highly engaged employee may be at risk of burning out. By suggesting a model that incorporates the complexity of the relationship between engagement and burnout as well as indicates that work engagement may be positive only to a certain point, after which it may increase the risk of emotional exhaustion and cynicism, our study adds to theory on engagement and burnout. Still, our findings should not be misinterpreted; that is, our study results support engagement theory and previous findings that engagement is mainly a good thing (e.g., [2]), although not necessarily always as our results indicate.

The results did not support our hypothesis concerning professional inefficacy; the finding was significant but had an unexpected curvature (i.e., U-shaped downward pattern). This finding may imply that being engaged at work provides a kind of illusion in the form of high perceived efficacy (e.g., “I am performing well”). This is likely because being highly engaged allows employees to demonstrate their personal dedication, efficacy, and energy, and thereby imagine that they can utilize their full potential at work [99]. The illusion may generate a delayed response between work engagement and professional inefficacy, which may explain our findings. Future research should investigate whether such an illusion is evident.

A second contribution of our study to work engagement theory concerns the need to consider the context in which work engagement occurs [6,7,31]. Our study contributes by clarifying the moderating roles of the mastery and performance climates. Our finding that employees who perceived high mastery climate levels were less prone to experiencing cynicism may indicate that employees are better able to sustain their engagement while maintaining the availability of their resources in high perceived mastery climate conditions. Given the emphasis on development, learning, growth, and cooperation, a mastery climate may mitigate the possibility of engaged employees becoming indifferent or distant in their attitudes toward work. This implies that individuals tend to respond to their social environments (e.g., motivational climates) differently when it comes to work engagement. An interesting question for future research would be to look into not only the extent to which individuals engage but also the ways in which they engage. Consistent with AGT, the current results indicate that individuals may have a healthier way of engaging when they are working in a mastery climate.

In our study, the buffering influence of a mastery climate became weaker for employees with medium-to-high levels of work engagement. This may indicate that, although mastery climate criteria are evident and help prevent resource loss, the influence of climate may differ according to the level of work engagement. In contrast to individuals with low-to-medium work engagement, highly engaged individuals may be less responsive to their social environments. A potential explanation could be that highly engaged individuals are so absorbed in their work that they are less able to take in social stimuli. This plausible explanation may relate to study findings on gamers where their increasing absorption in a game has been found to decrease their ability to take in stimuli from the “real world” [100]. Another possible explanation may be that the highly engaged employees are simply intrinsically motivated; that is, they invest in work because it is volitional and personally enjoyable or interesting. With such an autonomous self-regulation, they may be less responsive to the social milieu because they commit to their work fully without depleting their internal resources [101].

Further, in our study, employees with low work engagement reported higher cynicism in a high-performance climate than in a low-performance climate. This may indicate that a performance climate can enhance employees’ use of cynicism as a defense mechanism to protect themselves from exposure to stressful interpersonal or work-related situations [97]. Individuals in high-performance climates may be more likely to develop cynicism as a dysfunctional way to cope at work [102]. The drawback of developing such a coping strategy is the likelihood of perceiving even higher job demands and subsequently higher levels of exhaustion [103].

Given that that a perceived performance climate negatively moderated the linear relationships between work engagement and emotional exhaustion and between work engagement and cynicism, such a climate may amplify employees’ vulnerability to burnout. This finding is aligned with AGT and related research that has shown that burnout vulnerability is enhanced when individual mastery orientation is low and when the motivational climate dictates that successful achievement is accomplished through the demonstration of ability [56]. In addition, Lemyre, Treasure, and Roberts [104] found that elite swimmers became more susceptible to burnout over the course of a season as their motivation became less self-determined. Consistent with AGT and self-determination theory [105], such a shift from more to less autonomous motivational regulation can be evoked by performance climate criteria [104]. Thus, a performance climate may increase burnout risk because it does not support autonomy and because it facilitates competition and lack of support among employees.

### 5.2. Practical Implications

Our study has a few notable practical implications. First, having engaged employees may result in positive individual and organizational outcomes [2,3]. However, in line with our results, when engagement is excessively high, which is over the mid-level indicated in our study, employees may be at risk of developing burnout. Organizations and leaders are therefore advised to pay attention to their expectations of highly engaged employees. Two potential ways to accomplish this would be to encourage a healthy balance between work, home, and leisure activities and to restrict the amount of overtime worked. Identifying those employees who are highly engaged would also be important so as to avoid assigning them too many additional tasks and extra-role assignments. Leaders may view overtime work and the delivery of more than what is expected as positive outcomes, but our results indicate that managers should avoid exploiting engagement as a way to “motivate without money” [12] (p. 51). A mastery climate does not seem to be a sufficient buffer for excessively highly engaged employees. Nevertheless, the facilitation of a mastery climate is important to balance the detrimental aspects of a performance climate [34]. This can be accomplished by providing practices, such as job autonomy, task variation and meaningfulness, skill development, learning, supportive supervision, and valuing of employee/team effort and cooperation [19]. 

A performance climate seems to enhance employees’ vulnerability towards cynicism. Given that cynicism is believed to reduce the energy employees have available for performing work and for developing creative solutions to work problems [106], a performance climate may not be optimal for the facilitation of a healthy organization. Although many organizations encourage competitive behavior in order to obtain better results [32,107], we suggest that organizations and their leaders should consider the maladaptive aspects of such a climate. Leader behavior plays a particularly important role because it has a signaling function in that employees use the messages leaders send to make sense of their work situations and what is expected of them [108]. 

### 5.3. Limitations and Suggestions for Future Research

One of our study’s strengths is that it is based on a two-wave design. Still, our study is not without limitations. First, although our sample size was quite large, the initial response rate was only 25%. It is difficult to ascertain why the response rate was so low. Our union representative noted that the union had also sent out several other surveys to its members; thus, the union members may have been fatigued by all these surveys, making them less likely to respond. Although we controlled for the variables that significantly differed among respondents and nonrespondents, our study’s results should be interpreted with special care.

Second, given that we collected data through a union we were only able to measure the respondents’ perceived motivational psychological climate. Whether the respondents’ perceptions of the climate are shared with all other employees in their work situations (i.e., organizational climate) is not possible to know with our data. This would have required a multilevel design with separate studies in each organization where the respondents work. Thus, our findings should be interpreted with this in mind and future studies that include work climate measures should possibly avoid collecting data through a union.

Third, as with all nonexperimental research, we cannot demonstrate causal relations between the variables we studied [109]. Therefore, experimental or longitudinal studies with three or more waves are essential for drawing causal inferences. 

Fourth, our study depends on self-reported data to measure work engagement, burnout, and the perceived motivational climate. These measures are subject to common method bias and inflated ratings with respect to, for example, social desirability and the respondents’ implicit theories [81]. Still, work engagement, burnout, and the motivational climate are all perceptual variables. In order to reduce potential common method variance (CMV), we emphasized, in the information letter to the respondents, how their anonymity would be protected. In addition, we decided to investigate CMV’s potential influence by conducting a Harman’s single-factor test, in which all multiple study items measured at T1 were included [110]. The results indicated an emergence of six separate factors (i.e., work engagement, emotional exhaustion, cynicism, professional inefficacy, mastery climate, and performance climate). In addition, we conducted CFA analyses on both T1 and T2 data, which also indicated that CMV was not likely to have influenced our results. We decided not to continue with additional CMV tests, because scarce evidence and a consensus on the effects of statistical detection and correction techniques from other statistical procedures remain to control for CMV [81,111,112]. Still, we realise that the readers of this article may have dissimilar viewpoints concerning CMV (i.e., CMV exists, CMV does not exist etc.) that are likely to impact their assessment of our results [111]. Our findings should therefore be interpreted accordingly.

Fifth, some of the standardized beta coefficients in the current study were significant but very low. Therefore, their meaningfulness may be questioned [113]. A large sample size typically results in significant coefficients representing the association between relevant variables, but it is up to the researcher to consider their meaningfulness [113]. Yet, even if a variable explains only a small percentage of the variance, it may still provide practical significance [114]. However, the interpretations of the results presented in our study should be viewed in light of this limitation.

Sixth, we have no way of knowing whether the over-engagement we speak of in this article in fact represents engaged workaholics (cf., [115]) as we did not include workaholism as a control variable. Engaged workaholics have been found to be driven by both autonomous and controlled motivation and are therefore simultaneously pulled toward and pushed away from their work [115]. A fruitful focus for future research could therefore be to account for whether over-engagement in the sense discussed in this study is at issue only for those individuals characterized as engaged workaholics.

The two motivational climate structures are suggested to be interdependent and are likely to operate more or less simultaneously [19,32]. Therefore, an interesting avenue for future research could be to investigate the interactive (multiplicative) role of work engagement, mastery climate, and performance climate in predicting burnout. Given that multiple work climates are likely to operate simultaneously in an organization, it may be interesting to investigate how climates with competing goals, such as performance and mastery climates, affect outcomes [116]. 

## 6. Conclusions

Our study represents important theoretical and practical implications by challenging common assumptions in the occupational health literature and providing an alternative explanation for the relationship between work engagement and burnout. We stress that being highly engaged at work may not be exclusively positive and that employees with too much work engagement may be exposed to a higher risk of burnout (i.e., emotional exhaustion and cynicism). Thus, it seems possible that engagement can go awry and lead to burnout. Furthermore, our results suggest that a mastery climate may mitigate—and that a performance climate may enhance—the likelihood that employees will become indifferent or distant in their attitudes toward work (i.e., cynicism). With these results, we extend the current occupational health psychology research on work engagement and burnout.

## Figures and Tables

**Table 1:** Descriptive statistics for key study variables (N_T1_ = 8,282; N_T2_ = 1,081).

Variables	Mean	*SD*	1	2	3	4	5	6	7	8	9	10	11	12	13	14	15	16
1. Gender	1.25	0.44	-															
2. Age	44.56	10.88	−0.14 **	-														
3. Education	3.03	0.50	0.07 **	−0.07 **	-													
4. Work hours	39.18	8.39	−0.15 **	−0.01	0.01	-												
5. Work engagement T1	4.07	1.10	0.00	0.09 **	0.02	0.06 **	**(0.93)**											
6. Work engagement T2	4.20	1.03	−0.02	0.09 **	0.00	0.08 *	0.71 **	**(0.94)**										
7. Emotional exhaustion T1	1.62	1.20	0.06 **	−0.06 **	0.02	0.02	−0.42 **	−0.39 **	**(0.87)**									
8. Emotional exhaustion T2	1.62	1.21	0.05	−0.07 **	0.06	0.02	−0.42 **	−0.48 **	0.73 **	**(0.88)**								
9. Cynicism T1	1.44	1.08	−0.03 **	−0.04 **	0.00	0.01	−0.54 **	−0.46 **	0.53 **	0.44 **	**(0.82)**							
10. Cynicism T2	1.89	0.95	−0.06	−0.03	0.03	−0.01	−0.47 **	−0.58 **	0.40 **	0.54 **	0.65 **	**(0.82)**						
11. Professional inefficacy T1	1.42	0.89	−0.07 **	−0.08 **	−0.04 **	−0.03 **	−0.54 **	−0.41 **	0.26 **	0.26 **	0.38 **	0.38 **	**(0.79)**					
12. Professional inefficacy T2	1.87	0.71	−0.07 *	−0.02	−0.06	−0.05	−0.43 **	−0.51 **	0.24 **	0.30 **	0.34 **	0.43 **	0.58 **	**(0.78)**				
13. Mastery climate T1	3.56	0.78	0.03 *	0.06 **	0.00	0.01	−0.44 **	0.38 **	−0.31 **	−0.31 **	−0.43 **	−0.39 **	−0.28 **	−0.27 **	**(0.85)**			
14. Mastery climate T2	3.68	0.78	0.03	0.06	0.02	0.01	−0.40 **	0.51 **	−0.28 **	−0.34 **	−0.41 **	−0.52 **	−0.31 **	−0.35 **	0.69 **	**(0.87)**		
15. Performance climate T1	1.98	0.68	−0.07 **	0.04 **	0.00	0.07 **	−0.12 **	−0.09 **	0.26	0.21 **	0.26 **	0.22 **	0.11 **	0.07 *	−0.24 **	−0.22 **	**(0.83)**	
16. Performance climate T2	1.94	0.68	−0.09 **	−0.00	0.01	0.12 **	−0.10 **	−0.13 **	0.17	0.26 **	0.22 **	0.31 **	0.06 *	0.09 **	−0.21***	−0.25 **	0.66 **	**(0.84)**

*Notes.* T1 = Time 1; T2 = Time 2; Gender: 1 = male and 2 = female; Education: 1 = high school, 2 = vocational school, 3 = college, 4 = a university degree, and 5 = other. Cronbach’s α values for each measure are presented on the diagonal in parentheses and in bold. * *p* < 0.05. ** *p* < 0.01.

**Table 2:** The moderating role of the motivational climate (performance and mastery climates).

Variables	Emotional Exhaustion				Cynicism				Professional Inefficacy			
	Model 0	Model 1	Model 2	Model 3	Model 0	Model 1	Model 2	Model 3	Model 0	Model 1	Model 2	Model 3
Intercept	2.44 ** (0.13)	1.95 ** (0.12)	2.03 ** (0.11)	2.03 ** (0.11)	2.56 ** (0.13)	1.91 ** (0.11)	1.94 ** (0.11)	1.96 ** (0.11)	3.35 ** (0.10)	2.92 ** (0.08)	2.93 ** (0.08)	2.93 ** (0.08)
Gender	0.15 ** (0.03)	0.17 ** (0.03)	0.21 ** (0.03)	0.20 ** (0.03)	−0.11 ** (0.03)	−0.09 ** (0.03)	−0.04 (0.03)	−0.04 (0.03)	−0.17 ** (0.02)	−0.14 ** (0.02)	−0.13 ** (0.02)	−0.13 ** (0.02)
Age	−0.01 ** (0.00)	−0.00 (0.00)	−0.00 (0.00)	−0.00 (0.00)	−0.01 ** (0.00)	0.00 (0.00)	0.00 (0.00)	0.00 (0.00)	−0.01 ** (0.00)	−0.00 ** (0.00)	−0.00 ** (0.00)	−0.00 ** (0.00)
Education	0.03 (0.03)	0.05 * (0.02)	0.05 * (0.02)	0.05 * (0.02)	0.01 (0.03)	0.04 (0.02)	0.03 (0.02)	0.03 (0.02)	−0.07 ** (0.02)	−0.04 ** (0.01)	−0.05 ** (0.02)	−0.05 ** (0.02)
Work hours	0.00 * (0.00)	0.01 ** (0.00)	0.01 ** (0.00)	0.01 ** (0.00)	0.00 (0.00)	0.01 ** (0.00)	0.00 ** (0.00)	0.00 ** (0.00)	−0.00 ** (0.00)	−0.00 (0.00)	−0.00 (0.00)	−0.00 (0.00)
Work engagement (ENG)		−0.43 ** (0.01)	−0.35 ** (0.01)	−0.35 ** (0.01)		−0.56 ** (0.01)	−0.46 ** (0.01)	−0.46 ** (0.01)		−0.45 ** (0.01)	−0.43 ** (0.01)	−0.43 ** (0.01)
ENG^2^		0.03 ** (0.01)	0.04 ** (0.01)	0.03 ** (0.01)		0.05 ** (0.01)	0.05 ** (0.01)	0.05 ** (0.01)		−0.02 ** (0.00)	−0.01 * (0.01)	−0.02 ** (0.00)
Mastery climate (MC)			−0.16 ** (0.02)	−0.16 ** (0.02)			−0.35 ** (0.02)	−0.31 ** (0.02)			−0.05 ** (0.01)	−0.04 ** (0.01)
Performance climate (PC)			0.34 ** (0.02)	0.32 ** (0.02)			0.28 ** (0.02)	0.31 ** (0.02)			0.04 ** (0.01)	0.07 ** (0.01)
ENG × MC			0.01 (0.01)				0.08 ** (0.01)				−0.03 * (0.01)	
ENG^2^ × MC			0.01 (0.01)				0.03 ** (0.01)				0.00 (0.01)	
ENG × PC				−0.07 ** (0.02)				−0.08 ** (0.02)				0.02 (0.01)
ENG^2^ × PC				0.01 (0.01)				−0.02 ** (0.01)				−0.02 ** (0.01)
Deviance (χ^2^)	27,697.08	26,064.81	25,484.29	25,455.28	28,266.09	25,306.05	24,385.79	24,403.86	22,574.78	19,701.33	19,659.25	19,646.68
Decrease in deviance (Δχ^2 a^)		1632.27 **	580.52 **	609.53 **		2960.04 **	920.26 **	902.19 **		2873.45 **	42.08 **	54.65 **

*Notes. NT1* = 8282; *NT2* = 1081. Gender: 1 = male and 2 = female; Education: 1 = high school, 2 = vocational school, 3 = college, 4 = a university degree, and 5 = other. Estimated coefficients are displayed and standard errors are shown in parentheses. The outcomes emotional exhaustion, cynicism, and professional inefficacy reflect both Time 1 (T1) and Time 2 (T2), where the nested data take into account the variance of time (i.e., T1 and T2) as a control. The full ML estimator was used to calculate this decrease in deviance (Δχ^2^), which can be considered a way of expressing effect size in multilevel modeling. * *p* < 0.05. ** *p* < 0.01.
